# Intracellular receptor EPAC regulates von Willebrand factor secretion from endothelial cells in a PI3K-/eNOS-dependent manner during inflammation

**DOI:** 10.1016/j.jbc.2021.101315

**Published:** 2021-10-20

**Authors:** Jie Xiao, Ben Zhang, Zhengchen Su, Yakun Liu, Thomas R. Shelite, Qing Chang, Yuan Qiu, Jiani Bei, Pingyuan Wang, Alexander Bukreyev, Lynn Soong, Yang Jin, Thomas Ksiazek, Angelo Gaitas, Shannan L. Rossi, Jia Zhou, Michael Laposata, Tais B. Saito, Bin Gong

**Affiliations:** 1 Department of Pathology, University of Texas Medical Branch, Galveston, Texas, USA; 2 Department of Internal Medicine, Infectious Diseases, University of Texas Medical Branch, Galveston, Texas, USA; 3 Department of Pharmacology and Toxicology, University of Texas Medical Branch, Galveston, Texas, USA; 4 Department of Microbiology and Immunology, University of Texas Medical Branch, Galveston, Texas, USA; 5 Division of Pulmonary and Critical Care Medicine, Department of Medicine, Boston University Medical Campus, Boston, Massachusetts, USA; 6 The Estelle and Daniel Maggin Department of Neurology, Icahn School of Medicine at Mount Sinai, New York, New York, USA

**Keywords:** von Willebrand factor secretion, EPAC, endothelial cell, PI3K, eNOS, inflammation, Weibel–Palade body, AFM, spatial proximity, AFM, atomic force microscopy, BMECs, brain microvascular ECs, ECs, endothelial cells, eNOS, endothelial nitric oxide synthase, EPAC, exchange protein directly activated by cAMP, HUVECs, human umbilical vein endothelial cells, IF, immunofluorescence, iNOS, inducible nitric oxide synthase, LPS, lipopolysaccharide, NO, nitric oxide, PLA, proximity ligation assay, rTNFα, recombinant TNFα, TNFα, tumor necrosis factor-α, vWF, von Willebrand factor, WPBs, Weibel–Palade bodies.

## Abstract

Coagulopathy is associated with both inflammation and infection, including infections with novel severe acute respiratory syndrome coronavirus-2, the causative agent Coagulopathy is associated with both inflammation and infection, including infection with novel severe acute respiratory syndrome coronavirus-2, the causative agent of COVID-19. Clot formation is promoted *via* cAMP-mediated secretion of von Willebrand factor (vWF), which fine-tunes the process of hemostasis. The exchange protein directly activated by cAMP (EPAC) is a ubiquitously expressed intracellular cAMP receptor that plays a regulatory role in suppressing inflammation. To assess whether EPAC could regulate vWF release during inflammation, we utilized our *EPAC1*-null mouse model and revealed increased secretion of vWF in endotoxemic mice in the absence of the EPAC1 gene. Pharmacological inhibition of EPAC1 *in vitro* mimicked the *EPAC1*-/- phenotype. In addition, EPAC1 regulated tumor necrosis factor-α–triggered vWF secretion from human umbilical vein endothelial cells in a manner dependent upon inflammatory effector molecules PI3K and endothelial nitric oxide synthase. Furthermore, EPAC1 activation reduced inflammation-triggered vWF release, both *in vivo* and *in vitro.* Our data delineate a novel regulatory role for EPAC1 in vWF secretion and shed light on the potential development of new strategies to control thrombosis during inflammation.

Coagulopathy is associated with both severe inflammation and infections, including the novel severe acute respiratory syndrome coronavirus-2, the causative agent of COVID-19 (1, 2, 3, 4, 5). To maintain vascular patency, vascular endothelial cells (ECs) adjust the balance between blood coagulation, bleeding, and fibrinolysis on their luminal surfaces *via* complicated mechanisms (6, 7, 8, 9, 10, 11, 12, 13, 14, 15, 16). Accumulating evidence has suggested an extensive cross-talk between coagulation and inflammation, whereby inflammation leads to activation of coagulation that also considerably affects inflammatory activity (17, 18, 19). Systemic infectious diseases can activate ECs and propagate immune responses and inflammation in blood vessels, increasing the risk of microthrombosis, which has been documented in lethal and nonlethal COVID-19 cases (1, 2, 3, 4, 5).

The membrane-associated, adhesive glycoprotein von Willebrand factor (vWF) is synthesized in ECs and megakaryocytes, and its main function is to directly promote clot formation by capturing platelets and chaperoning clotting factor VIII (20, 21, 22). Current research has confirmed that vWF is both a plasma glycoprotein known for its role in blood clotting and a modulator of inflammatory responses (23, 24, 25, 26, 27). A fundamental mechanism that ECs use to regulate coagulation is by secreting vWF in elongated secretory organelles known as Weibel–Palade bodies (WPBs) (21, 22, 28, 29). The two principal protein constituents of WPBs are vWF and P-selectin, but WPBs contain a variety of other molecules involved in inflammation (29) and intercellular communication (30), including angiopoietin-2 (25), IL-8 (31), and eotaxin-3 (31), and the intracellular/extracellular vesicle membrane protein CD63 (30). vWF can determine the assembly of WPBs, and WPB egress is associated with the secretion of vWF and P-selectin from ECs in response to various stimuli (6, 22, 32). P-selectin is a neutrophil and monocyte adhesion molecule important in the initiation of inflammation (18, 33). Collectively, regulating endothelial secretion of vWF from WBPs may modulate not only thromboembolic formation but also inflammation.

Endothelial secretion of vWF from WBPs is under tight control (20, 34, 35). In general, endothelial WPBs become responsive to exogenous stimuli that increase intracellular calcium levels or the second messenger cAMP (36, 37, 38). The effects of cAMP are transduced by two ubiquitously expressed intracellular cAMP receptors, PKA and exchange protein directly activated by cAMP (EPAC). EPAC proteins are a family of intracellular sensors for cAMP. In mammals, the EPAC protein family contains two members: EPAC1 and EPAC2 (39, 40). Both EPAC isoforms function by responding to increased intracellular cAMP levels in a PKA-independent manner and act on the same immediate downstream effectors, the small G proteins Rap1 and Rap2 (40, 41). EPAC1 is the major isoform in ECs. Rap activation by EPAC1, but not EPAC2, contributes to the effects of cAMP-elevating hormones on endothelial barrier functions (42, 43, 44). Growing evidence has revealed that the cAMP–EPAC signaling axis plays a regulatory role in suppressing inflammation (45, 46). The first identified non-cAMP EPAC1-specific agonist, I942, was shown to suppress IL-6 signaling and inflammatory gene expression in ECs in response to inflammatory stimuli (46), suggesting EPAC1 plays an endothelial function and stabilizing role during inflammation (43, 47). In ECs, it has been documented that cAMP provokes the secretion of vWF *via* the cAMP–PKA pathway (29, 35). We have reported that cAMP–EPAC complexes are involved in hemostasis by driving endothelial luminal surface expression of tissue plasminogen activator receptor annexin A2, thereby promoting vascular fibrinolysis both *in vivo* and *in vitro* (7). However, whether cAMP–EPAC is involved in regulating endothelial vWF secretion during inflammation remains to be elucidated. In contrast to the documented EC function-stabilizing effects of the EPACs (43, 47), an *in vitro* study (48) showed that human umbilical vein endothelial cells (HUVECs) increase release of vWF after a short exposure to an EPAC-specific cAMP analog, which can be hydrolyzed by serum esterases (49, 50) and requires starvation media to work (51).

In the present study, taking advantage of our *EPAC1* KO mouse model (7), the EPAC-specific inhibitor NY173 (52), and the non-cAMP EPAC1-specific agonist I942 (51), we defined the role(s) of EPAC1 in the regulation of vWF secretion from ECs in response to inflammatory stimuli both *in vivo* and *in vitro*. We found that *EPAC1* gene deletion elevated the secretion of vWF in endotoxemic mice. Inactivation of EPAC1 potentiated tumor necrosis factor-α (TNFα)-triggered vWF release. I942 had the capability to reduce the secretion of vWF during inflammation both *in vivo* and *in vitro*. Mechanistic studies indicated that activation of host PI3K/endothelial nitric oxide synthase (eNOS) can attenuate the efficacy of the EPAC-specific inhibitor and limit vWF secretion during inflammation.

## Results

### Inactivation of EPAC1 increases vWF secretion and contributes to the progression of microthrombi in LPS-treated *EPAC1*-KO mice

**Figure 1:**
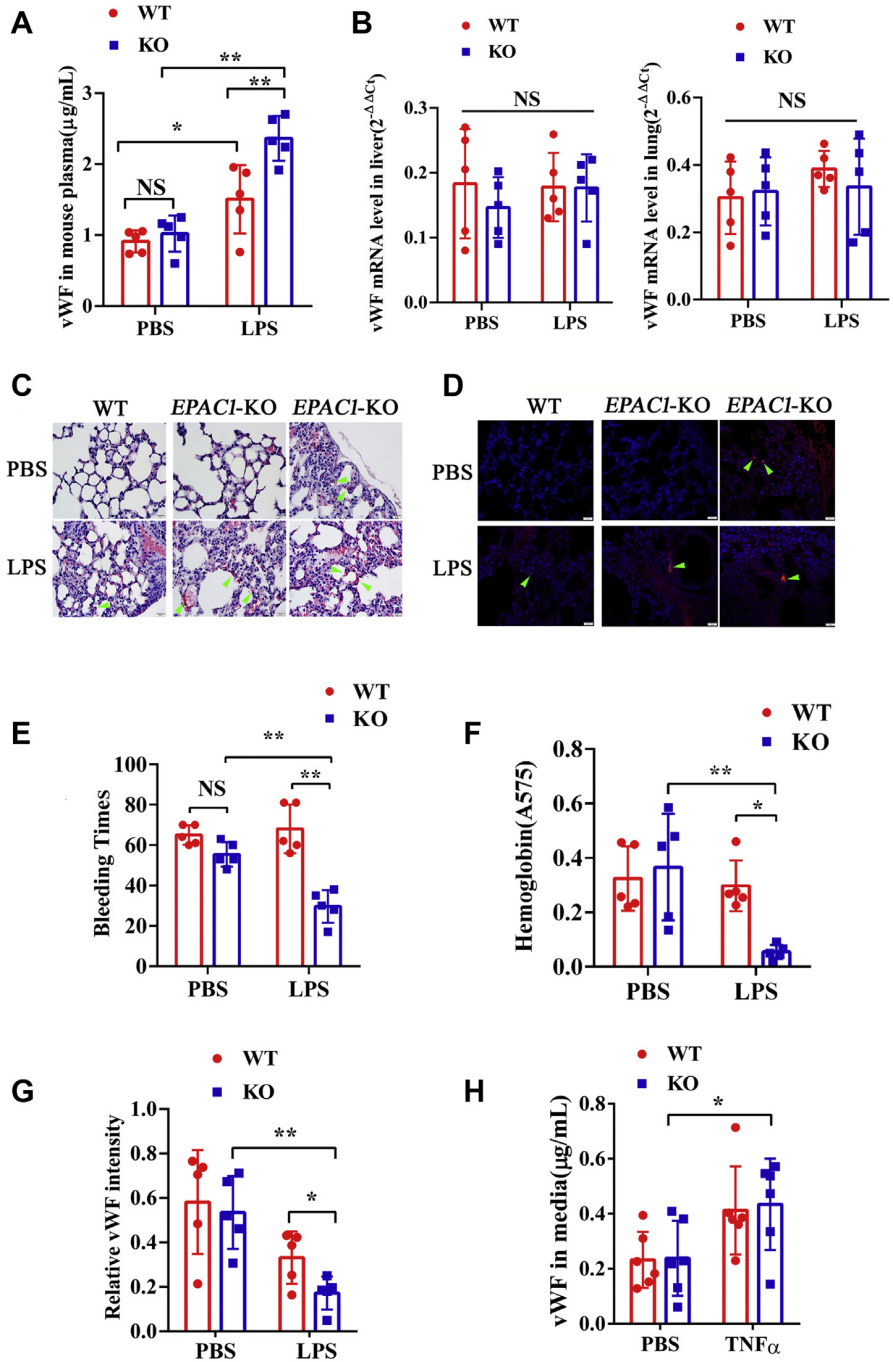
**Inactivation of EPAC1 increases vWF secretion and enhances microthrombosis during inflammation**. Plasma vWF concentrations and vWF mRNA expression in the tissues of WT and *EPAC1*-KO mice treated with or without lipopolysaccharides (LPS). *A*, the plasma levels of vWF between the WT and *EPAC1*-KO mice in the presence or absence of LPS (5 mg/kg/d, i.p) for 2 h. n = 5 for each group. *B*, qRT-PCR analysis of vWF mRNA expression in the lungs and livers of WT and *EPAC1*-KO mice treated with or without LPS. Two-way ANOVAs showed that the differences in vWF mRNA expression was not significant. n = 5 for each group. *C* and *D*, the formation of microthrombi in the microvessels of LPS-treated *EPAC1*-KO mice. WT (*A* and *B*) and *EPAC1*-KO (*C–F*) mice were treated with PBS (*A* and *C*) or LPS (5 mg/kg/d, i.p. ×1). (*B*, *D–F*). After 24 h, the lungs were dissected after euthanasia and whole-animal perfusion. The lungs were immersion-fixed in 10% buffered formalin overnight. Microthrombi were detected in all LPS-treated *EPAC1*-KO mice (*D–F*). The scale bars indicate 20 μm. *D*, IF of fibrin(ogen) were performed in the lungs. *E*, the bleeding time of the WT and *EPAC1*-KO mice treated with LPS. The LPS-treated *EPAC1*-KO mice have decreased tail bleeding times. *F*, blood loss quantified as the amount of hemoglobin (absorbance at 575 nm) released during the tail bleeding test in the LPS-treated WT and *EPAC1*-KO mice, ∗*p* < 0.05. n = 5 for each group (*E* and *F*). *G*, the relative intensity of immunofluorescence signals of vWF in lung sections from WT *versus EPAC1*-KO mice, which were thoroughly perfused with PBS and analyzed using ImageJ software. The data are expressed as the ratios of immunofluorescence intensities of vWF normalized against DAPI signals (55). n = 5 for each group. *H*, the vWF concentrations in the culture medium of WT *versus EPAC1*-KO mouse aortic ECs. n = 6 mice for each group. ∗*p* < 0.05 and ∗∗*p* < 0.01. The scale bars represent 20 μm. DAPI, 4′,6-diamidino-2-phenylindole; ECs, endothelial cells; EPAC, exchange protein directly activated by cAMP; IF, immunofluorescence; LPS, lipopolysaccharides; ns, not significant; qRT-PCR, reverse transcription quantitative PCR; TNFα, tumor necrosis factor-α; vWF, von Willebrand factor.

In testing the constitutive plasma levels of vWF, we observed no difference between WT and *EPAC1*-KO mice (Fig. 1*A*). The difference in vWF mRNA expression between these two mouse strains was not significant (Fig. 1*B*). To further explore the EPAC1 function on inflammation-triggered vWF expression and secretion *in vivo*, WT and *EPAC1*-KO mice were treated with lipopolysaccharide (LPS) (5 mg/kg/d, i.p.) or PBS for 2 h. The results showed that exposure to LPS increased the plasma levels of vWF; compared with their LPS-treated WT counterparts, the plasma levels of vWF were higher in LPS-treated *EPAC1*-KO mice (*p* < 0.01) (Fig. 1*A*). However, the change in vWF mRNA expression in lung and liver tissues was not significant (Fig. 1*B*). LPS-treated *EPAC1*-KO mice showed thrombosis, and microthrombi were detected in the lungs of LPS-treated *EPAC1*-KO mice but were almost absent in LPS-treated WT mice (Table 1, Figures 1, *C* and *D*, and Fig. S1*A*).

**Table 1:** Detection of microthrombi in mice

Group	Number of animals	Score of microthrombi^a^				Total
		3	2	1	0	
PBS*-*treated *EPAC1*-KO mice	5	0	0	0	5	0^b^
PBS*-*treated WT mice	5	0	0	0	5	0
LPS-treated *EPAC1*-KO mice	5	0	0	5	0	5
LPS-treated WT mice	5	0	0	1	4	1^b^

a The left lungs were collected for RNA extraction, and the right lungs were fixed for histopathology studies. Tissue sections of the lungs, livers, and brains were stained with H&E. For animals in which thrombi were detected in the lungs by H&E staining using 100× power microscopy, sections were further validated with immunofluorescence staining against fibrin using the rabbit anti-mouse fibrin(ogen) polyclonal antibody as described (7). Score of 3: microthrombi could be detected in any sections of the above three organs; score of 2: microthrombi could be detected in any sections of the above two organs; score of 1: microthrombi could be detected in only one section of the above organs; score of 0: no microthrombi could be detected in any sections of the above organs. No thrombus was detected in the livers and brains by H&E staining.

b *p* < 0.01, compared with the LPS-treated *EPAC1*-KO group.

Because vWF performs critical functions in primary hemostasis, a tail bleeding time test was conducted (53). The bleeding time of LPS-treated *EPAC1*-KO mice was shorter than that of LPS-treated WT mice (Fig. 1*E*). The total blood loss, assessed by its hemoglobin content, corresponded closely with the observed bleeding time of LPS-treated *EPAC1*-KO and WT mice (Fig. 1*F*). Prothrombin time and activated partial thromboplastin time were also detected. There was no significant difference between the groups (Fig. S2). These results suggested that EPAC1 controls the primary hemostasis of endotoxemic mice through altering the concentration of vWF in the plasma. However, variations in prothrombin time and activated partial thromboplastin time, which are routine coagulation screening tests for secondary hemostasis, were not observed in this study.

Furthermore, using a published method (54) to quantify expression levels of vWF in the lungs that are PBS-perfused, we observed that deletion of *EPAC1* enhanced LPS treatment–triggered reduction of vWF levels in lung tissues (Fig. 1*G*). Moreover, we observed that treatment with recombinant TNFα (rTNFα) (50 ng/ml) increased vWF secretion from *EPAC1*-KO mouse aortic ECs *in vitro* (*p* < 0.05), whereas there was no statistical difference between the WT-PBS and WT-rTNFα groups (*p* = 0.17) (Fig. 1*H*).

In conclusion, *EPAC1*-KO mice show a higher level of plasma vWF than WT mice in a state of inflammation in which the progression of microthrombi is detectable.

### A pharmacological inhibitor of EPAC increases vWF secretion during inflammation in ECs

**Figure 2:**
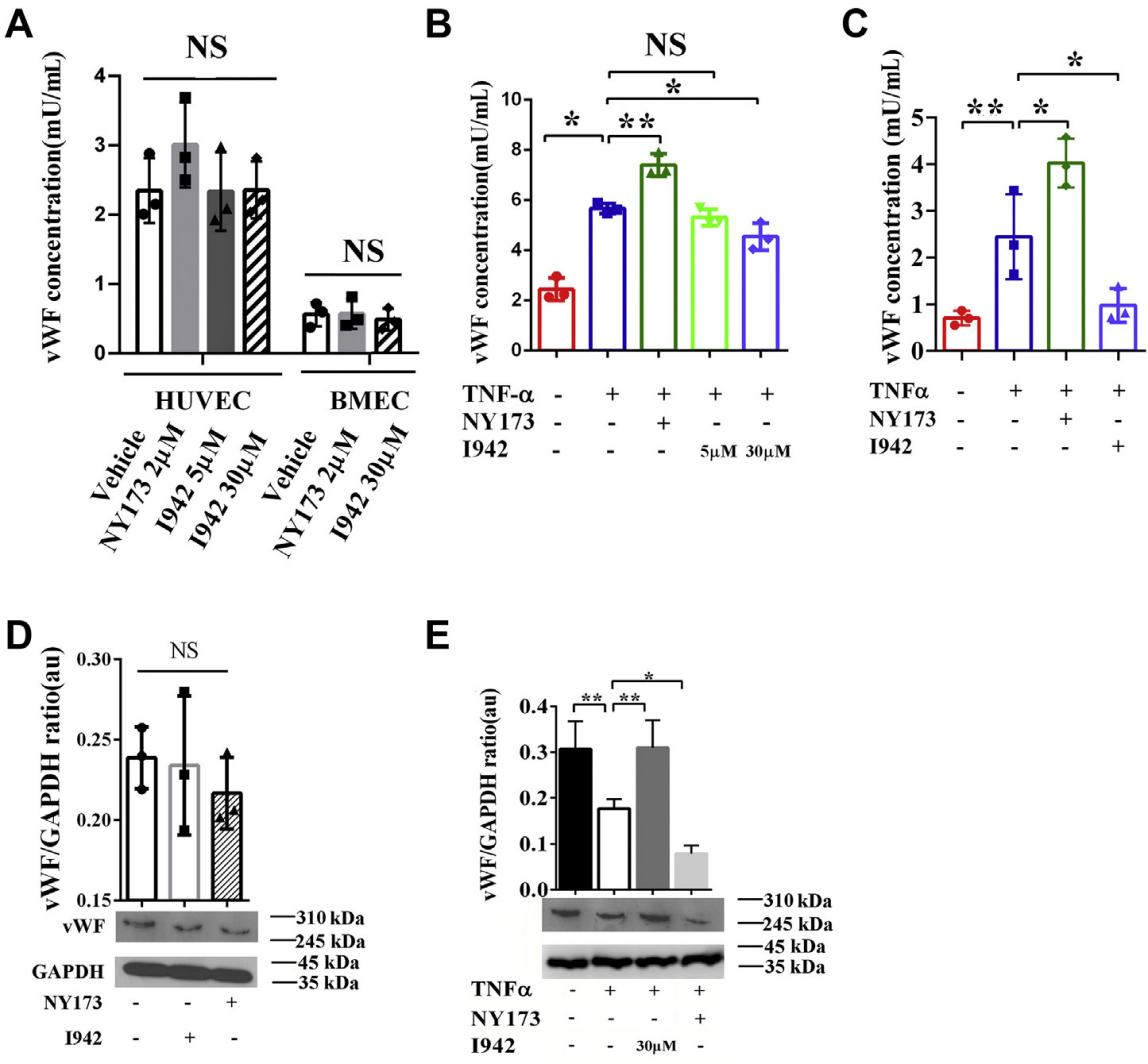
**Pharmacological manipulations of EPAC1 modulate the inflammation-triggered vWF secretion from HUVECs**. *A*, the effect of only NY173 or I942 on vWF secretion. HUVECs and BMECs were incubated with I942 (5 or 30 μM) or NY173 (2 μM) for 24 h. The vWF concentrations in the culture medium from ECs were detected using the human vWF ELISA kit. n = 3 for each group. *B*, the vWF concentrations in the culture medium of HUVECs. NY173 enhanced the secretion of vWF from rTNFα-treated HUVECs. The EPAC1-specific agonist I942 at 30 μM, but not 5 μM, significantly reduced the secretion of vWF from rTNFα-treated HUVECs. *C*, the vWF concentrations in the culture medium of BMECs. *D*, the vWF protein levels in the cell lysates of HUVECs incubated with I942 (30 μM) or NY173 (2 μM) for 24 h. *E*, the vWF protein levels in the cell lysates of HUVECs. vWF expression was significantly reduced in rTNFα-treated HUVECs. The vWF protein expression was further significantly decreased in the NY173 + rTNFα group. I942 (30 μM) significantly increased vWF expression in rTNFα-treated HUVECs. n = 3 for each group. ∗*p* < 0.05 and ∗∗*p* < 0.01. au, arbitrary units of the ratio of vWF to GAPDH; BMECs, brain microvascular ECs; ECs, endothelial cells; EPAC, exchange protein directly activated by cAMP; HUVECs, human umbilical vein endothelial cells; NS, not significant; TNFα, tumor necrosis factor α; rTNFα, recombinant TNFα; vWF, von Willebrand factor.

Although the detailed mechanism underlying LPS-triggered enhanced secretion of vWF *in vivo* remains unclear, TNFα has been characterized as a pivotal mediator of endotoxin shock (55) and can regulate vWF expression, being widely used experimentally as an inflammatory stimulus for vWF secretion (56). In this study, we chose TNFα as a stimulator of inflammation *in vitro*. We observed that vWF levels were increased in the culture media of HUVECs and brain microvascular ECs (BMECs) treated with rTNFα (50 ng/ml) for 4 h (*p* < 0.05) (Fig. 2, *A* and *B*). To determine the effect of EPAC1 inhibition on EC secretion of vWF in response to inflammatory mediators, we treated HUVECs and BMECs with NY173, a novel EPAC-specific inhibitor (52) to inhibit EPAC1 in HUVECs. NY173 did not affect the viability of HUVECs and BMECs when the concentration was 1 μM to 5 μM (Fig. S3, *A* and *B*). NY173 (2 μM) alone had little impact on vWF secretion from HUVECs (Fig. 2*A*) and intracellular vWF expression in HUVECs (Fig. 2*D*). We found that pretreatment with NY173 (2 μM) for 24 h enhanced rTNFα-triggered vWF secretion into media (*p* < 0.01) (Fig. 2, *B* and *C*). Western immunoblotting was used to detect intracellular vWF protein expression, which was reduced in the rTNFα-treated group of HUVECs (*p* < 0.05) and more significantly reduced in the NY173 + rTNFα-treated group, *versus* the rTNF-only–treated group (*p* < 0.01) (Fig. 2*E*).

### Pharmacological activator of EPAC1 decreases inflammation-triggered vWF secretion from ECs

The EPAC1-specific agonist I942 is the first identified noncyclic nucleotide small molecule with agonist properties toward EPAC1, but I942 shows very little agonist action toward EPAC2 or PKA (57, 58). Concentrations of I942 ranging from 5 μM to 50 μM did not affect the viability of HUVECs and BMECs (Fig. S3, *A* and *B*). I942 (30 μM) had no effect on vWF secretion from HUVECs and BMECs (Fig. 2*A*). Western immunoblotting results showed that I942 (30 μM) had little influence on intracellular vWF expression in HUVECs (Fig. 2*D*). In this study, HUVECs were pretreated with I942 (30 μM) for 24 h before exposure to rTNFα (50 ng/ml) for 4 h. I942 (30 μM) downregulated the secretion of vWF from rTNFα-treated HUVECs and BMECs (*p* < 0.05) (Fig. 2, *B* and *C*). Intracellular vWF protein expression, which was reduced in HUVECS treated with rTNFα (*p* < 0.05), was significantly elevated in the I942 + rTNFα–treated group compared with the rTNFα-only group (*p* < 0.01) (Fig. 2*E*). Combined with the result that the EPAC-specific inhibitor NY173 upregulates rTNFα-induced vWF release, these data demonstrate that EPAC1 modulates vWF secretion from HUVECs and a non-cAMP, EPAC1-specific agonist (I942) has the capacity to limit vWF secretion during inflammation.

### EPAC1 modulates the exocytosis of WPBs during inflammation

**Figure 3:**
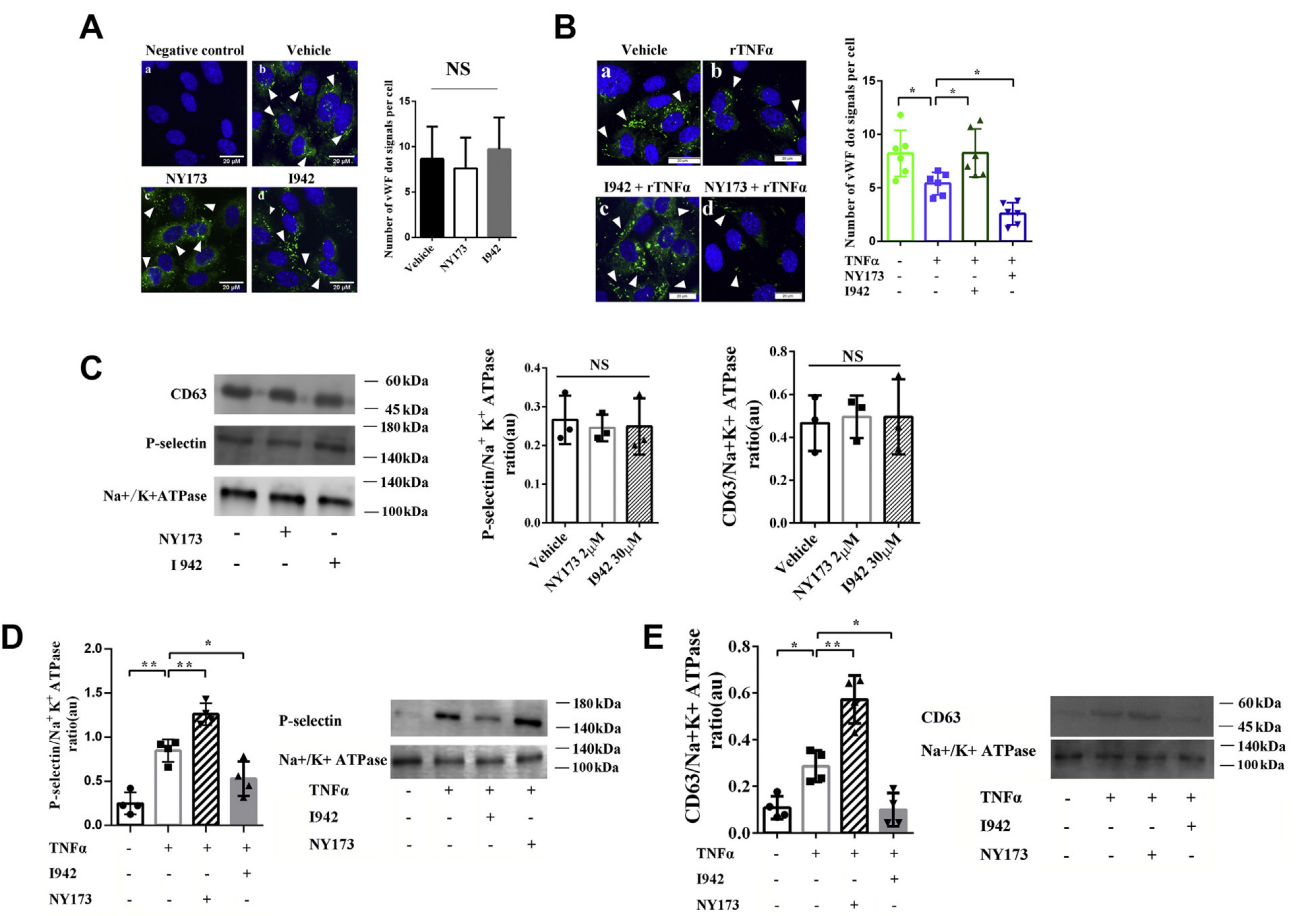
**EPAC1 modulates the exocytosis of WPBs during inflammation.***A*, immunofluorescence of vWF in HUVECs in the noninflammation state: (*a*) negative control where normal mouse IgGs were used as primary antibodies, (*b*) vehicle-only treated cells, (*c*) HUVECs treated with 2 μM NY173 for 24 h, and (*d*) HUVECs treated with 30 μM I942 for 24 h. In *b*, *c*, and *d*, the samples were incubated with the anti-vWF mouse monoclonal antibody as the primary antibody and then stained with DAPI (*blue*) and Alexa Fluor 488–conjugated secondary antibody for vWF labeling (*green*). The scale bars indicate 20 μm. The quantitative analyses of vWF-positive puncta and cell nuclei were performed using ImageJ software. The results are expressed as dot signals enumerated in each cell. Five microscopic fields were examined for each sample. The results are expressed as dot signals enumerated in each cell. n  =  3 for each group. *B*, immunofluorescence of vWF in HUVECs in the inflammation state. The HUVECs were pretreated with NY173 or I942 for 24 h and treated with rTNFα (50 ng/ml) for 4 h: (*a*) vehicle only, (*b*) rTNFα only, (*c*) I942 + rTNFα, (*d*) NY173 + rTNFα. The scale bars represent 20 μm. ImageJ was used to quantify vWF-positive puncta. Five microscopic fields were examined for each case. The results were expressed as dot signals enumerated in each cell. n = 6 for each group (*A* and *B*). *C*, Western immunoblotting was performed to analyze the expression of CD63, P-selectin, and the sodium–potassium pump in the membrane fractions of HUVECs. The graphs of the relative ratios in arbitrary units (au) of P-selectin or CD63 with Na^+^/K^+^-ATPase are shown. n = 3 for each group. *D*, Western immunoblotting analysis of the expression of P-selectin in the membrane fractions of HUVECs treated with rTNFα only, NY173 or I942, and rTNFα, and untreated. A graph of the relative ratio in au of P-selectin with Na^+^/K^+^-ATPase is shown. *E*, Western immunoblotting analysis of the expression of CD63 in the membrane fractions of HUVECs treated with NY173 or I942 alone with rTNFα. A graph of the relative ratio in au of CD63 with Na^+^/K^+^-ATPase is shown. n = 4 for each group (*D* and *E*). ∗*p* < 0.05 and ∗∗*p* < 0.01. EPAC1, exchange protein directly activated by cAMP; DAPI, 4′,6-diamidino-2-phenylindole; HUVECs, human umbilical vein endothelial cells; IgG, immunoglobulin G; NS, not significant; rTNFα, recombinant TNFα; TNFα, tumor necrosis factor; WPBs, Weibel–Palade bodies; vWF, von Willebrand factor.

To examine the effect of EPAC1 on WPB secretion during inflammation, vWF immunofluorescence (IF) microscopy was used to detect intracellular WPBs (59, 60). The IF imaging of WPBs reveled rod or dot shapes (arrowheads in Fig. 3*A*). HUVECs were first treated with NY173 (2 μM) or I942 (30 μM) for 24 h. Data obtained using ImageJ analyses showed that the difference in the number of WPBs among the vehicle-only, NY173-only, and I942-only groups was not significant. Next, HUVECs were pretreated with NY173 (2 μM) or I942 (30 μM) for 24 h followed by rTNFα (50 ng/ml) for 4 h. ImageJ calculations revealed that the number of WPBs in the rTNFα group was significantly decreased, suggesting that WPB release could be triggered by rTNFα (Fig. 3*B*). However, I942 limits the rTNFα-stimulated exocytosis of WPBs, whereas the EPAC-specific inhibitor NY173 enhances such egress (Fig. 3*B*).

In addition to vWF, P-selectin and CD63 are detected in WPBs (61). In this study, HUVECs were treated with 2 μM NY173 or 30 μM I942 for 24 h. The expressions of P-selectin and CD63 in plasma membranes of HUVECs did not change significantly (Fig. 3*C*). To further assess whether WPB exocytosis during inflammation can be modulated by EPAC1, HUVECs were treated with NY173 or I942 for 24 h followed by rTNFα (50 ng/ml) for 4 h. Increased expression of P-selectin in the membrane fractions from cells treated with rTNFα + NY173 was observed compared with rTNFα-only–treated groups (Fig. 3*D*). Similar results were observed for the expression of CD63 (Fig. 3*E*). Conversely, the EPAC1-specific agonist I942 decreased P-selectin and CD63 expressions in the membrane fractions from rTNFα + I942–treated cells (Fig. 3, *D* and *E*).

**Figure 4:**
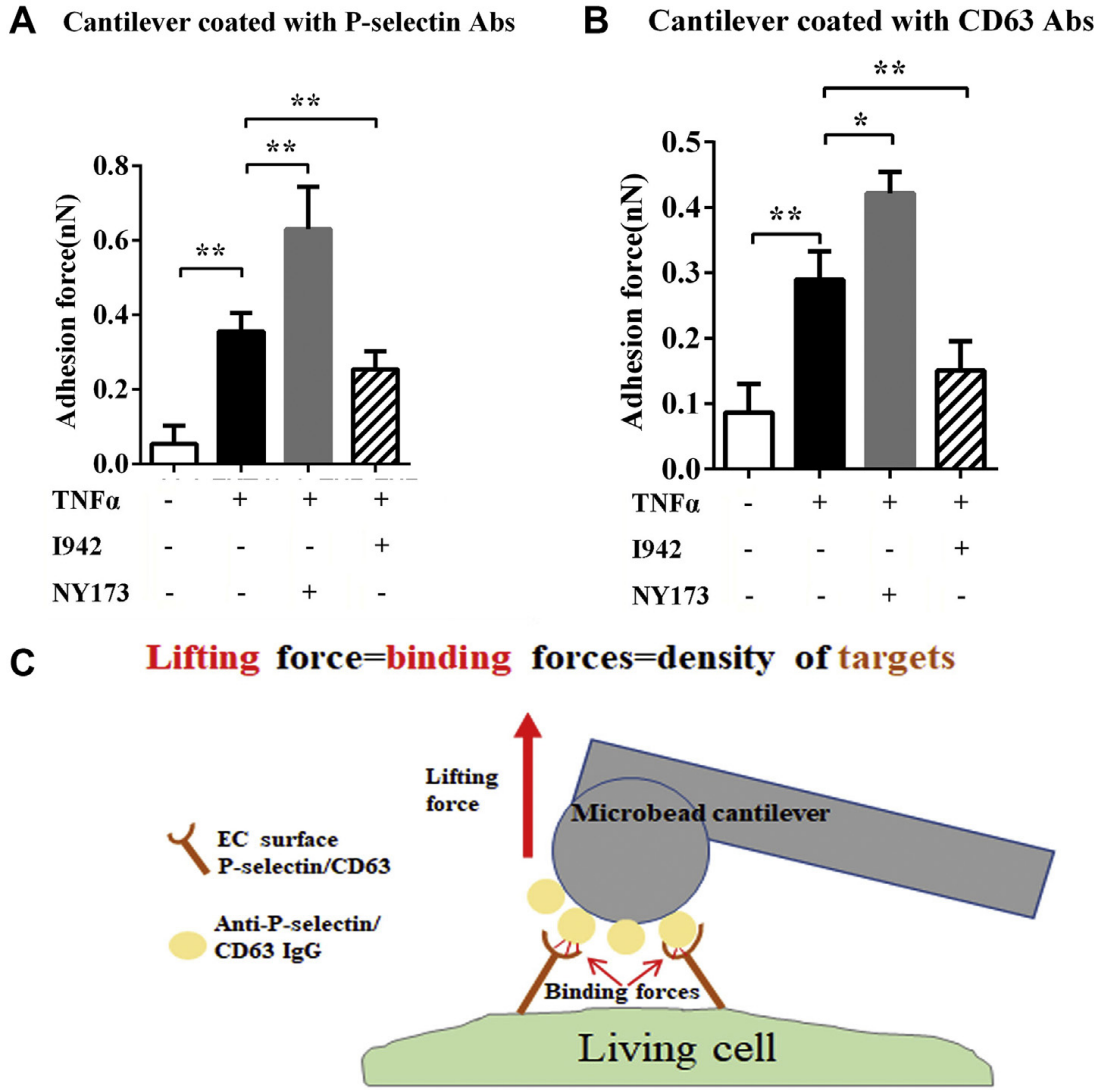
**EPAC1 affects P-selection and CD63 residing on endothelial apical surfaces**. Quantification of P-selectin (*A*) and CD63 (*B*) expression on the surface of live HUVECs as measured by atomic force microscopy (AFM). A normal mouse IgG-coated colloidal cantilever was used during calibration of the AFM system before measuring the unbinding forces between an anti-P-selectin– or anti-CD63 antibody–coated cantilever and the cell (for details, see Experimental procedures). With either anti-P-selectin– or anti-CD63 antibody–coated cantilevers, the adhesion forces were significantly stronger in the NY173 + TNFα–treated HUVECs than the TNFα-only–treated groups; ∗*p* < 0.05 and ∗∗*p* < 0.01. n = 12 for each group. *C*, a schematic depicting the specific unbinding force measurements that can be compiled for quantification of total P-selectin or CD63 expression on the surface areas of a live HUVEC. AFM, atomic force microscopy; EC, endothelial cell; HUVECs, human umbilical vein endothelial cells; IgG, immunoglobulin G; TNFα, tumor necrosis factor-α.

To further validate these results in single living cells, atomic force microscopy (AFM) was used to quantify the distribution of CD63 and P-selectin on the surface of HUVECs. AFM has been used to determine the expression levels of cell surface proteins by measuring the binding affinity of specific protein–protein interactions with nanoforce spectroscopy (7). In the present study, the specific unbinding force was measured during rupture of the interaction between the antigen (CD63 or P-selectin) expressed at the apical surface of live HUVECs and the antibody-coated AFM cantilever probe. Interactions between antibodies on the AFM cantilever and cell surface antigens cause large adhesion forces, which are quantified by the deflection signal during separation of the cantilever from the cell (Fig. 4). The results showed that adhesion forces in the NY173 + rTNFα–treated group were stronger than those in the rTNFα-only group; the adhesion forces in the I942 + rTNFα–treated group were weaker than those in the rTNFα-only group (Fig. 4).

These data provide strong evidence that pharmacological inhibition or activation of EPAC1 affects the expression of P-selectin and CD63 on cell membranes and modulates the inflammatory-triggered exocytosis of WPBs in HUVECs.

### EPAC1 manipulates the spatial proximity between P-selectin and vWF in HUVECs

**Figure 5:**
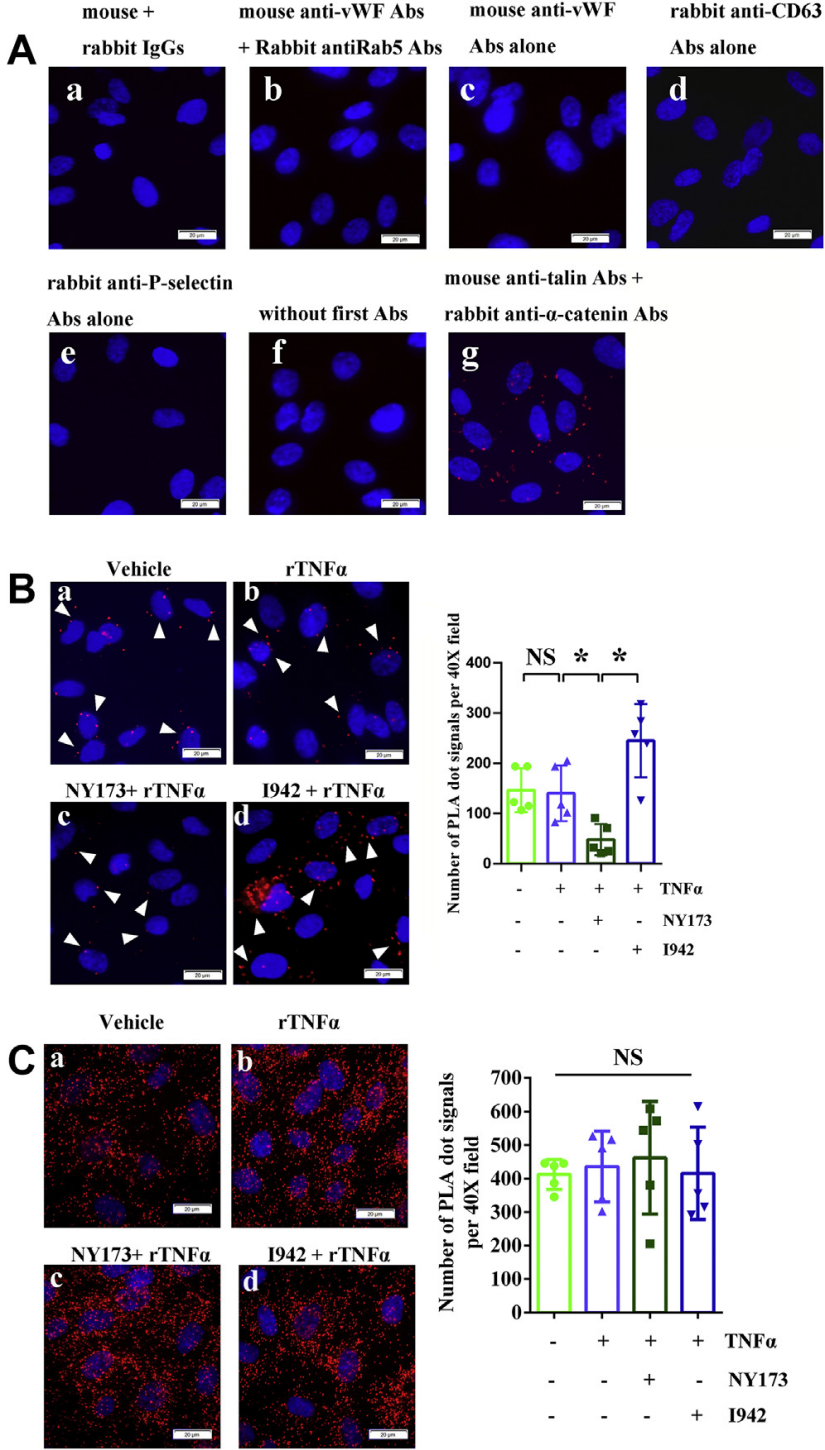
**PLA signals of vWF-CD63 and vWF-P-selectin in HUVECs**. *A*, control groups: (*a*) reagent-negative control where normal mouse and rabbit IgGs were used as primary Abs, (*b*) negative control where mouse anti-vWF Abs and rabbit anti-Rab5 Abs were used as primary Abs, (*c*) mouse anti-vWF Abs alone, (*d*) rabbit anti-CD63 Abs alone, (*e*) rabbit anti-P-selectin Abs alone, (*f*) no first Abs, and (*g*) positive control where mouse anti-talin Abs and rabbit anti-α-catenin were used as primary Abs (*red*). *B* and *C*, IFA staining: the representative PLA signals (*red*) of vWF-P-selectin (*B*, *arrowheads*) and vWF-CD63 (*C*), respectively, in HUVECs. (*a*) vehicle only, (*b*) rTNFα, (*c*) NY173 pretreatment + rTNFα, and (*d*) I942 pretreatment + rTNFα. The nuclei in panels *A–C* were counterstained with DAPI (*blue*). The scale bars indicate 20 μm. The graphs show the quantitative analysis of vWF-P-selectin PLA signals (*B*) and vWF-CD63 PLA signals (*C*) using ImageJ software. Five 40× microscopic fields were examined for each case. The results are expressed as dot signals enumerated in each 40× field. n  = 5 per group. ∗*p* < 0.05. Abs, antibodies; DAPI, 4′,6-diamidino-2-phenylindole; HUVECs, human umbilical vein endothelial cells; IFA, immunofluorescence assay; IgG, immunoglobulin G; NS, not significant; PLA, proximity ligation assay; rTNFα, recombinant tumor necrosis factor-α; vWF, von Willebrand factor.

We next assessed whether P-selectin is merely part of the molecular cargo of WPBs or whether it is closely correlated with vWF in ECs. Previous studies have shown that P-selectin can bind to D′-D3 domains of vWF, which is crucial for P-selectin recruitment (62). Moreover, D′-D3 domains of vWF have already been implicated in vWF storage and multimerization (63, 64). These studies suggest that P-selectin is more than a cargo protein of WPBs. Understanding the complex molecular spatial relationship of protein–protein physical interactions is essential for understanding their functional outcome (65). In this study, the proximity ligation assay (PLA) was used to detect P-selectin–vWF spatial proximities and CD63–vWF spatial proximities in HUVECs during inflammation, as we described (65). Both P-selectin–vWF proximities and CD63–vWF proximities could be detected in HUVECs (Fig. 5). More importantly, pharmacological manipulation of EPAC1 regulates P-selectin–vWF proximities (Fig. 5*B*), but not CD63–vWF proximities (Fig. 5*C*). Signals detected by PLAs of P-selectin–vWF in HUVECs were increased in the I942-treated group, whereas NY173 induces the opposite effect (Fig. 5*B*). The results of the PLAs suggest that protein–protein proximal interactions exist between vWF and P-selectin or CD63 and that the P-selectin–vWF spatial proximity can be modulated by EPAC1.

### EPAC1 regulates TNFα-triggered vWF secretion in a PI3K- or eNOS-dependent manner

**Figure 6:**
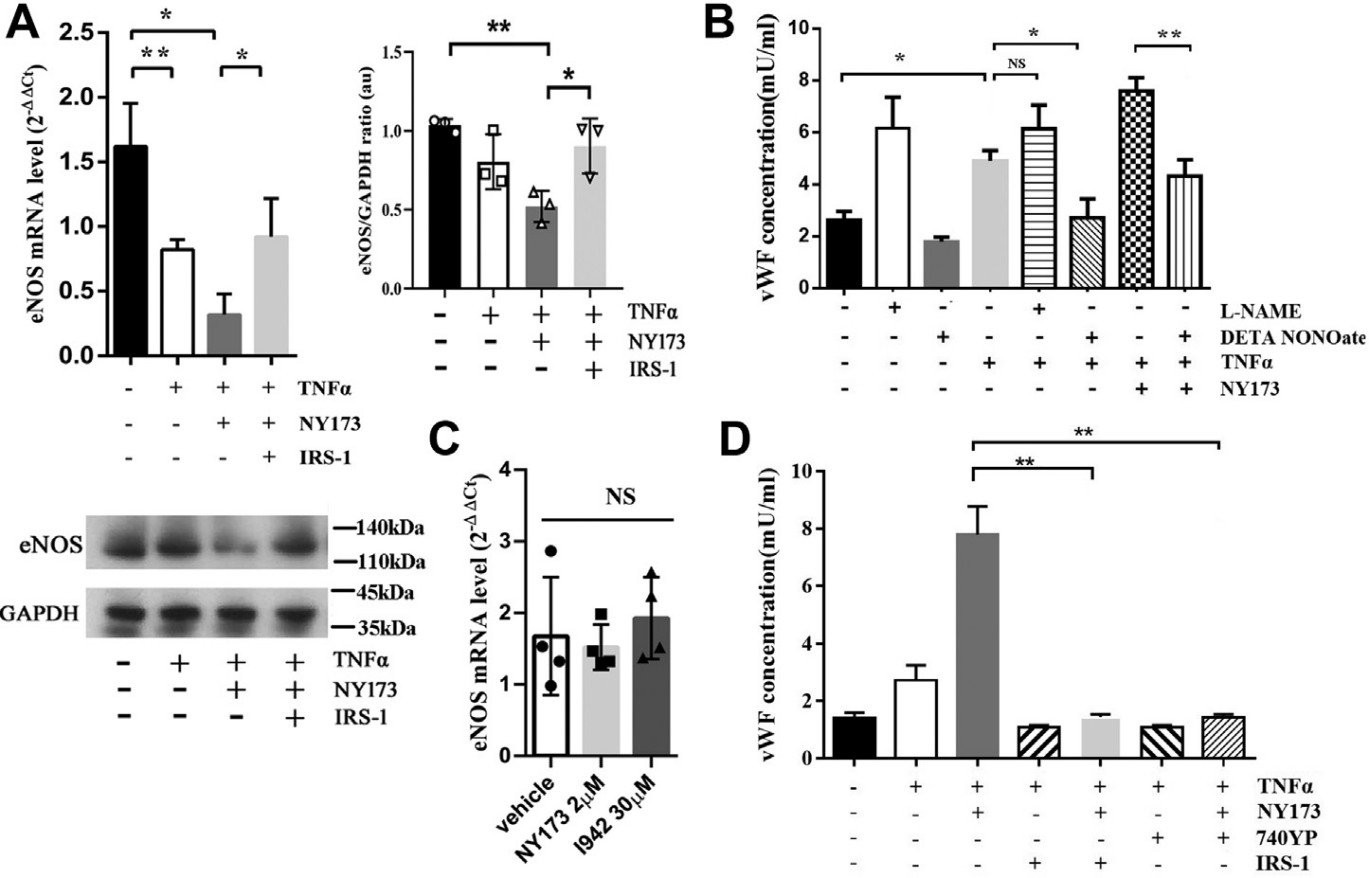
**EPAC regulates the vWF secretion by HUVECs in a PI3K-/eNOS-dependent manner**. *A*, RT-qPCR analysis of eNOS mRNA expression in HUVECs and a representative protein immunoblot of eNOS. The eNOS mRNA expression decreased in the HUVECs treated with rTNFα for 4 h; in the presence of NY173 for 24 h and rTNFα for 4 h, eNOS mRNA expression further declined. The PI3K activator IRS-1 (with rTNFα treatment) reversed the effect of NY173 on mRNA expression. The immunoblot displayed that in the presence of NY173 and rTNFα, eNOS expression was suppressed, whereas adding IRS-1 reversed the effect of NY173. *B*, the assessment of eNOS on vWF secretion triggered by rTNFα as measured by ELISA. The data show that DETA NONOate significantly downregulated rTNFα-induced vWF secretion. The regulatory role of EPAC on rTNFα-triggered vWF secretion was eNOS-dependent. *C*, the effect of NY173 and I942 on eNOS mRNA expression in HUVECs, without exposure to TNFα. The HUVECs were incubated with 2 μM NY173 or 30 μM I942 for 24 h. *D*, the assessment by ELISA of the PI3K effect on EPAC-regulated vWF secretion by HUVECs. The vWF concentrations in culture media were detected in all groups: vehicle-only, rTNFα-only, rTNFα + NY173, rTNFα + IRIS-1, rTNFα + NY173 + IRIS-1, rTNFα +740YP, and r-TNFα + NY173 + 740YP–pretreated HUVECs. n = 3 for each group. ∗*p* < 0.05 and ∗∗*p* < 0.01. eNOS, endothelial nitric oxide synthase; EPAC, exchange protein directly activated by cAMP; HUVECs, human umbilical vein endothelial cells; NS, not significant; qRT-PCR, reverse transcription quantitative PCR; rTNFα, recombinant TNFα; TNFα, tumor necrosis factor-α; vWF, von Willebrand factor.

The nitric oxide (NO) system has a wide range of biological properties to maintain vascular homeostasis. The TNFα effect on NO messaging is associated with downregulation of eNOS (66, 67), and the lack of eNSO likely contributes to the release of vWF (68). We therefore examined whether rTNFα decreased eNOS expression, and whether it then promoted vWF release from HUVECs. We found that rTNFα reduced the eNOS mRNA but not inducible nitric oxide synthase (iNOS) mRNA levels in HUVECs (*p* < 0.01) (Fig. 6*A* and Fig. S4). The NOS inhibitor L-NAME hydrochloride could enhance rTNFα-induced vWF secretion in HUVEC culture media, but the results were not statistically significant (*p* = 0.64) between the rTNFα-only and the rTNFα + L-NAME groups. Nevertheless, rTNFα-triggered vWF secretion could be markedly reversed by DETA NONOate, an NO donor (Fig. 6*B*). These results suggested that TNFα promotes vWF secretion through downregulating the expression of eNOS in HUVECs.

Next, we examined whether the regulatory role of EPAC on rTNFα-triggered vWF secretion was eNOS-dependent, by comparing pretreatment with NY173 only or together with an NO donor before challenge with rTNFα. RT-qPCR showed that neither NY173 (2 μM) nor I942 (30 μM) by themselves affected the mRNA level of eNOS (Fig. 6*C*). However, the eNOS mRNA level was further decreased in the NY173 + rTNFα group compared with the rTNFα-only group (*p* < 0.05) (Fig. 6*A*). No difference in iNOS mRNA levels was observed among these groups (Fig. S4). Furthermore, the effect of NY173 on TNFα-triggered vWF secretion can be neutralized by an NO donor (*p* < 0.01) (Fig. 6*B*).

Activation of EPAC leads to PI3K-dependent PKB activation (69). The EPAC–PI3K–eNOS signaling pathway may serve as a downstream pathway of adenylyl cyclases (70). To evaluate the potential effect of the EPAC–PI3K–eNOS signaling pathway on vWF secretion during inflammation, we incubated NY173-pretreated HUVECs with or without the PI3K-specific activator IRS-1 (71) before exposure to rTNFα. In the presence of IRS-1, eNOS mRNA transcription (Fig. 6*A*), but not iNOS transcription (Fig. S4), was markedly increased. Furthermore, activation of PI3K in NY173-pretreated HUVECs significantly reduced rTNFα-triggered vWF secretion compared with the NY173-treated group that did not receive IRS-1 (Fig. 6*D*). Another PI3K activator, 740YP (10 μM) (72), had a similar effect (Fig. 6*D*). These results suggest that EPAC1 regulates inflammation-triggered vWF secretion in a PI3K- or eNOS-dependent manner.

### The EPAC1-specific agonist I942 reduces LPS-induced vWF secretion *in vivo*

**Figure 7:**
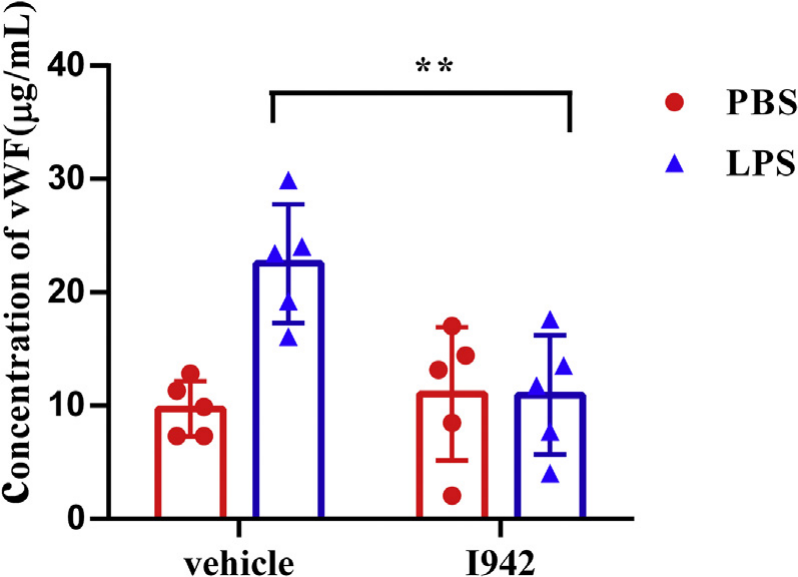
EPAC1-specific agonist I942 reduces LPS-induced vWF secretion in the WT mice. ELISAs were performed to analyze the plasma vWF concentrations in the C57BL/6 mice that were treated with I942. I942 was administrated at 5 mg/kg/day intraperitoneally before the mouse was exposed to LPS (5 mg/kg, i.p.) or equal volume of PBS for 2 h. I942 downregulated the plasma vWF levels in endotoxemic mice, ∗∗*p* < 0.01. n = 5 for each group. EPAC, exchange protein directly activated by cAMP; LPS, lipopolysaccharides; vWF, von Willebrand factor.

To further evaluate the effects of EPAC1 on vWF secretion during inflammation *in vivo*, WT mice were pretreated with I942 (5 mg/kg/d, i.p. ×3) or PBS, followed by LPS injection (5 mg/kg/d, i.p. ×1) or PBS. ELISAs were performed to detect plasma vWF concentrations. The results showed that I942 treatment significantly reduced plasma vWF levels in endotoxemic mice (n = 5) compared with the control group (n = 5) (Fig. 7), suggesting that targeting EPAC1 with an EPAC1-specific agonist can potentially control coagulopathy during inflammation.

## Discussion

Using *in vivo* and *in vitro* models, we demonstrated that EPAC1, as a documented EC function–stabilizing effector, regulates vWF secretion from ECs triggered by inflammation. Deletion of the *EPAC1* gene or pharmacological inactivation of EPAC1 significantly facilitated inflammation-induced vWF secretion. Inversely, activation of EPAC1 by an EPAC1-specific agonist (I942) attenuated vWF secretion in ECs during inflammation. An EPAC-specific inhibitor (NY173) increased P-selectin expression in the membrane fractions and affected the protein spatial proximity of P-selectin–vWF. Activation of PI3K or increased NO expression in NY173-treated HUVECs significantly reduced the efficacy of NY173 in promoting inflammation-triggered vWF secretion. Furthermore, by utilizing a mouse model of endotoxemia, we observed that an EPAC1-specific agonist (I942) reduced inflammation-induced vWF secretion *in vivo*. These *in vivo* and *in vitro* data help unravel a novel regulatory role for EPAC1 in vWF secretion by ECs. Importantly, the cAMP–EPAC signaling axis can be exploited as a potential target for the development of compounds to control vWF secretion that is triggered by inflammation.

The hallmark of acute and chronic inflammation is the widespread activation of ECs that provokes excessive vWF secretion from the storage pool of ECs by WPB exocytosis (73). vWF is secreted *via* three pathways—regulated secretion, basal secretion, and constitutive secretion (22, 34). The first two types of vWF-secretion pathways occur from WPBs and deliver highly multimerized vWF, but the third pathway (constitutive secretion) releases vWF that has not been packaged into WPBs and thus has not undergone high levels of multimerization (38). In contrast to continuously secreting vWF from the basal secretion pathway, the release of vWF from the regulated pathway occurs only after stimulation of ECs with an appropriate agonist, providing the endothelium with the means to react to its microenvironment by finely tuning the rate of release. Thus, controlling WPB exocytosis from ECs is an important way to regulate the concentration of vWF in plasma during inflammatory stress (73, 74). Vascular leakage syndrome can be accompanied by microthrombi (75). *In vitro* evidence suggests that EPAC1 controls vascular endothelial–cadherin–mediated cell junction formation (76, 77, 78). An *in vivo* study showed that deletion of *EPAC1* inhibits the endothelial barrier baseline in the skin and intestine, but not the heart (79). We reported that, compared with WT mice, the Evans Blue assay revealed no differences in the baseline of extravascular dye in the brain and lung parenchyma in *EPAC1*-KO mice; IF analysis displayed similar structures of vascular tight or adherens junctions (7). Research to investigate the potential interplay between endothelial barrier dysfunction and formation of microthrombi is warranted for future mechanistic studies.

It has been proved that treatment of HUVECs with I942, which represents an effective tool to probe the function of cellular EPAC1, leads to alterations in the expression of a wide variety of genes associated with vascular function. Specifically, I942 suppresses expression of proinflammatory adhesion molecules in HUVECs (46). After an inflammatory trigger, ECs are activated, prompting the massive release of WPBs that contain vWF and P-selectin (80). We observed that rTNFα-induced vWF secretion was increased in the EPAC-specific inhibitor (NY173) pretreatment group, whereas the EPAC1-specific agonist (I942) had the opposite effect. In addition to these *in vitro* results, *EPAC1*-KO endotoxemic mice had higher levels of plasma vWF concentration than WT endotoxemic mice. The results confirm that EPAC1 plays an important role in regulating inflammation-triggered vWF secretion. In contrast to our study, van Hooren *et al.* (48) reported that 8-pCPT-2′-O-Me-cAMP-AM, an EPAC-specific cAMP analog, promotes the release of vWF in HUVECs. This discrepancy could be due to different reagents and experimental conditions used in their *in vitro* experiments. In van Hooren's study, HUVECs were incubated with serum-free medium and supplemented with 1 μM 8-pCPT-2′-O-Me-cAMP-AM for 60 min. cAMP analogs are hydrolyzed by serum esterases (49, 50) and require starvation media in which to work (51), which restricts applications using primary ECs (47, 81, 82). Moreover, evidence has been provided that most cAMP and cGMP analogs have multiple targets, including some EPAC-specific analogs (81, 82). In our study, we used the noncyclic nucleotide EPAC1-specific agonist I942 to treat HUVECs for 24 h followed by rTNFα (50 ng/ml) for 4 h with the complete medium. We observed not only that the EPAC1-specific agonist could inhibit vWF secretion triggered by inflammation but also that the EPAC-specific inhibitor NY173 increased inflammation-triggered vWF secretion, in accordance with our observations for our *in vivo* study using WT and *EPAC1*-KO mice. In our study, the tail bleeding times between WT and *EPAC1*-KO mice showed no differences. However, in Nygaard's study, EPAC1-deficient mice showed a prolonged bleeding phenotype (83). Although the procedure of the tail bleeding time test was almost the same and WT C57BL/6J mice were used as controls in the experiment, their results differ from ours; the average tail bleeding time in Nygaard's study is around 500 s in WT C57BL/6J mice and beyond 20 min in Epac1-deficient mice. In our study, the bleeding time was about 66 s in WT mice and 55 s in *EPAC1*-KO mice. One study found that the average tail bleeding time was 51 s in C57BL/6 mice (84), which is very close to our findings. Compared with other published experimental data (85), the mice tail bleeding times in Nygaard's study (83) were significantly longer.

P-selectin is a critical component of WPBs and is anchored to EC surfaces. Exocytosis of WPBs follows inflammatory stimulation of ECs, which then provide cell surface sites for P-selectin ligands to bind circulating leukocytes (86, 87). In the present study, P-selectin expression in the membrane fractions was elevated in rTNFα-treated HUVECs upon pretreatment with the EPAC-specific inhibitor NY173, whereas the expression was attenuated in that group upon pretreatment with the EPAC1-specific agonist I942. The expression of CD63, an essential cofactor to leukocyte recruitment by endothelial P-selectin (30), was consistent with the change in P-selectin expression. To our knowledge, this is the first report that EPAC regulates the expression of P-selectin and CD63 on the cell membrane after inflammatory stimulation. Our results confirm the well-documented EC function-stabilizing effects of EPACs (46). Moreover, we provide evidence that P-selectin, a plasma marker of endothelial damage and dysfunction, serves as more than just a cargo protein for WPBs. It has been reported that P-selectin binds to the D′-D3 domains of vWF, which are not only crucial for P-selectin recruitment but also are implicated in vWF storage (62, 63). In this study, PLAs were used to detect the spatial proximity between P-selectin and vWF, and results showed that pharmacological manipulation of EPAC1 can regulate P-selectin–vWF spatial proximities. The domains of vWF binding with P-selectin are critical to the storage of vWF. The functional role(s) of the P-selectin–vWF proximity and whether EPAC1 can manipulate the secretion of vWF by regulating the P-selectin–vWF proximity remain to be determined.

Under normal physiological conditions, ECs help prevent inflammation and inhibit clotting partly through the continuous production of NO (88, 89). Viral infections, such as severe acute respiratory syndrome coronavirus-2, cause the release of inflammatory cytokines, injure ECs, and cause a significant decline in NO production (90). TNFα can downregulate eNOS expression in ECs (66). Moreover, eNOS reduction leads to vWF release (68). In the present study, we also observed that rTNFα (50 ng/ml) incubated with HUVECs for 4 h could potently decrease eNOS mRNA expression. vWF secretion triggered by TNFα can be manipulated using an NOS inhibitor or NO donor to regulate NO expression in HUVECs. It has been reported that EPAC activation can enhance eNOS activity in ECs (91). Likewise, we observed that NY173 further decreased TNFα-induced eNOS mRNA reduction. The NO donor, in turn, counteracted vWF secretion from HUVECs after treatment with NY173 and TNFα.

PI3Ks have been linked to an extraordinarily diverse group of cellular functions, including inflammation and coagulation (92, 93, 94). An earlier study has shown that PI3K is crucial in mediating the prothrombotic potential of ECs (95). *In vivo* studies have shown that inhibition of PI3K strongly enhances activation of ECs, upregulates LPS-induced coagulation and inflammation, and reduces the survival time of mice (94). We incubated NY173-pretreated HUVECs with the PI3K-specific activator IRS-1 before challenge with rTNF-α. We found that IRS-1 could dramatically reverse the effect of NY173 by inhibiting the eNOS expression and release of vWF. Activation of PI3K in NY173-pretreated ECs significantly reduced the TNFα-induced inhibition of eNOS expression and vWF secretion compared with the NY173-pretreated group that did not receive IRS-1. In support of the conclusion that EPAC mediates eNOS expression through the PI3K–Akt pathway from Namkoong *et al.* (70), we hypothesize that EPAC may regulate rTNFα-triggered vWF release in a PI3K–eNOS–dependent manner. Further studies, such as the level of NO detection in the supernatants, will allow us to confirm our current findings and those reported by van Hoooren *et al.* (96) in which the PI3K inhibitor LY294002 reduced epinephrine-induced release of vWF. Comparing that study with ours, we found considerable differences in reagents and experimental conditions, including different timepoints observed in the two studies. The incubation time of epinephrine and LY294002 in van Hooren's study ranged from 10 to 50 min, whereas our incubation time using PI3K activators was 24 h followed with 4 h of rTNFα treatment. In addition, epinephrine was used in van Hooren's study to activate vWF secretion. As we know, the cAMP–EPAC pathway is a way that epinephrine stimulates vWF release (37, 48), and the cAMP–PKA pathway is another way that epinephrine regulates and affects vWF secretion (97, 98, 99). EPAC and PKA might mediate opposing effects on PKB. Activation of EPAC leads to PI3K-dependent PKB activation, whereas stimulation of PKA may inhibit PKB activity (69).

Despite significant progress in the development of cAMP analogs as EPAC agonists (100), the EPAC isoform selectivity of these cAMP analogs and their potential off-target effects on other molecules, such as cAMP phosphodiesterase, remain challenging (81, 82). The EPAC1-specific agonist, I942, is the first identified noncyclic nucleotide small molecule with agonist properties toward EPAC1, with very little agonist action toward EPAC2 or PKA (57, 58), and has the potential to suppress proinflammatory cytokine signaling. This reduces the risk of side effects associated with general cAMP-elevating agents that activate multiple response pathways in HUVECs (58). In this study, I942 displayed a capacity to exert its pharmacological effects in complete EC culture medium and it downregulated inflammation-triggered vWF secretion in HUVECs. Moreover, this report involving *in vivo* application of I942 shows that *in vivo* activation of EPAC1 significantly reduced plasma vWF concentrations in LPS-induced endotoxemic mice. Additional work is needed to further explore whether I942 can act as a novel therapeutic to regulate hemostasis.

**Figure 8:**
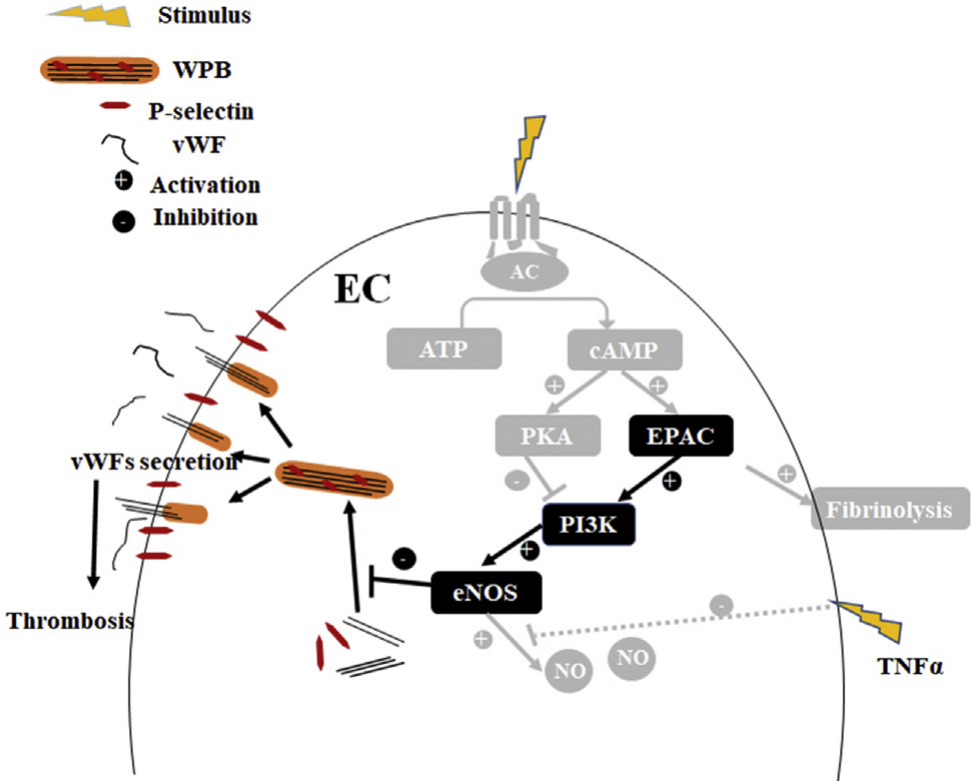
**Model for EPAC1 regulating inflammation-triggered vWF release in a PI3K-/eNOS-dependent manner.** Our proposed pathways from the present study are in *black*, and previously reported pathways are in *gray*. EC, endothelial cell; eNOS, endothelial nitric oxide synthase; EPAC, exchange protein directly activated by cAMP; NO, nitric oxide; TNFα, tumor necrosis factor-α; WPB, Weibel–Palade body; vWF, von Willebrand factor.

Taken together, this study combined an *in vitro* primary human endothelial system with an *in vivo* mouse model to reveal the role of EPAC in inflammation-triggered vWF secretion. In vascular ECs, EPAC1 regulates inflammation-triggered vWF release in PI3K- and eNOS-dependent manners (Fig. 8). The EPAC1-specific agonist I942 has the capacity to limit vWF secretion during inflammation *in vivo*. These data shed light on the potential development of new strategies to controlling the risk of thrombosis during inflammation.

## Experimental procedures

### Ethics statement

The mouse experiments performed for this study were carried out in accordance with National Institutes of Health, United States Department of Agriculture, and the Institutional Animal Care and Use Committee guidelines. The protocol supporting this study was approved by the Institutional Animal Care and Use Committee of the University of Texas Medical Branch.

### Endotoxemic mouse models induced by lipopolysaccharide

We used a mouse model of endotoxemia that consisted of intraperitoneal injection of a high dose of *Escherichia coli* LPS (5 mg/kg) (*E. coli* serotype O111:B4; Sigma). For more information, please see supporting information.

### AFM to measure cell surface expression of the target protein

As described previously (101), the biomechanical properties of P-selectin or CD63 at the cell surface were studied using an AFM system (Flex-AFM, Nanosurf AG) that utilized relevant antibody-functionalized AFM probes. For more information, please see supporting information.

### Statistics

Statistical significance was determined using a Student's *t* test or ANOVA. Results were regarded as significant if two-tailed *p* values were <0.05. All data are expressed as the mean ± SEM.

## Data availability

All data are included within the article or the supporting information.

## Supporting information

This article contains supporting information (7, 51, 52, 65, 94, 101, 102, 103, 104, 105, 106, 107, 108, 109, 110, 111, 112, 113).

## Conflict of interest

The authors declare that they have no conflicts of interest with the contents of this article.
