# Supplementary material for: Intracellular receptor EPAC regulates von Willebrand factor secretion from endothelial cells in a PI3K-/eNOS-dependent manner during inflammation
Source: J Biol Chem. 2021 Oct 20;297(5):101315. doi: 10.1016/j.jbc.2021.101315 (PMC8526113; doi:10.1016/j.jbc.2021.101315)

**Intracellular receptor EPAC regulates von Willebrand factor secretion from endothelial cells in a PI3K/eNOS-dependent manner during inflammation**

Jie Xiao^1, †^, Ben Zhang^1, †^, Zhengchen Su^1^, Yakun Liu^1^, Thomas R. Shelite^2^, Qing Chang^1^, Yuan Qiu^1^, Jiani Bei^1^, Pingyuan Wang^3^, Alexander Bukreyev^1^, Lynn Soong^4^, Yang Jin^5^, Thomas Ksiazek^1^, Angelo Gaitas^6^, Shannan L. Rossi^1^, Jia Zhou^3^, Michael Laposata^1^, Tais B. Saito^1,^ ^7^, Bin Gong^1, *^.

**Supporting Information**

**Materials and Methods**

**Cell culture**

HUVECs (human umbilical vein endothelial cells) (Cell Applications, Atlanta, GA) were cultured in Endothelial Cell Growth Medium (Cell Applications) with a humidity of 5% CO_2_ at 37°C. The media was changed every 48 hrs until cells reached 90% confluence. Cells passaged 2 to 5 times were used for all experiments. To stimulate HUVECs, the cells were cultured in media supplemented with 50 ng/mL recombinant TNFα (rTNFα, Millipore Sigma, Burlington, MA) for 4 hrs (1). Cells were then fixed using a 4% paraformaldehyde solution in phosphate-buffered saline (PBS) for 30 min at room temperature (RT) prior to further processing.

Mouse aortic endothelial cells (ECs) were isolated from six wild-type (WT) and six *Epac1* knockout (KO) mice as we described (2). Briefly, aortae were first dissected from mice and then cleaned of adipose tissue and cut into five rings per mouse aorta. A total of 30 aortic rings were cultured on Matrigel (Thermo Fisher Scientific, Waltham, MA) in Prigrow I media (Applied Biological Materials, Richmond, BC, Canada). Microscopic evidence of neo-cellular growth was presented after about one week in culture.

**Exchange protein directly activated by cAMP (EPAC) inhibitor and activator**

EPAC-specific inhibitor (NY173) and activator (I942) products were synthesized in Dr. Jia Zhou’s laboratory at the University of Texas Medical Branch (UTMB) (3,4). To determine the effects of NY173 and I942 on HUVEC viability *in vitro*, cytotoxicity was determined using the MTT Cell Proliferation Assay kit from Cayman Chemical (Ann Arbor, MI, USA) after culturing HUVECs with different concentrations of (1, 2, 3, or 5 μM) of NY173 and (5, 10, 30, or 50 μM) of I942, respectively, for 24 h. Mock-treated HUVECs served as controls. H_2_O_2_ (200 μmM)-treated HUVECs served as positive controls. The absorbance at 490 nm was recorded using a BioTek ELx808 plate reader (BioTek, Winooski, VT, USA). Results are presented as the mean ± standard error of the mean for three independent experiments; each experiment was conducted in triplicate. Based on unpublished pharmacokinetic and toxicology data from another ongoing project, I942 at 5 mg/kg/day (given by the intraperitoneal route) for three days induced no significant abnormalities in clinical signs and histology in normal mice.

**Special reagents**

The phosphoinositide 3-kinase (PI3K)-specific activator 740YP and the nitric oxide synthase (NOS) inhibitor L-NAME hydrochloride (L-NAME) were purchased from Tocris Bio-Techne (Minneapolis, MN, USA); the insulin receptor substrate (IRS-1 Tyr^608^) was purchased from Enzo Life Sciences, Inc. (Farmingdale, NY, USA); Nitric Oxide donor Diethyleneriamine NONOate (DETA NONOate) was from Cayman Chemical. For *in vitro* studies, the final concentration of special reagents in the HUVEC culture medium was as follows: NY173: 2 μM; I942: 30 μM; 740YP: 20 μM; IRS-1 Tyr^608^: 50 μg/L; DETA NONOate: 100 μM; and L-NAME: 100 μM. Medium with 0.01% dimethyl sulfoxide (vol/vol) was used as vehicle control.

**Plasma membrane protein isolation and cell fractionation**

Membrane protein isolation and cell fractionation were performed using the Minute™ Plasma Membrane Protein Isolation and Cell Fractionation Kit (Invent Biotechnologies, Eden Prairie, MN, USA), as we described (5,6). The resultant fractions were resuspended in RIPA buffer for immunoblotting to detect the expressions of P-selectin and CD63 in plasma membrane. Purity of the membrane fractions was verified by immunoblotting with antibody raised against membrane-specific (Na^+^/K^+^-ATPase ) proteins (6).

**Proximity ligation assay (PLA)**

The spatial proximities of P-selectin-vWF or CD63-vWF was determined using the Proximity Ligation Assay, as we described recently (7). Information for PLA-associated reagents is as follows (all reagents from Millipore Sigma): Duolink^®^ In Situ PLA^®^ probe donkey anti-rabbit IgG (H+L) (catalog #DUO92002), Duolink^®^ In Situ PLA^®^ probe donkey anti-mouse IgG (H+L) (catalog #DUO92004), Duolink^®^ In Situ Detection Reagents Red (catalog #DUO92008), Duolink^®^ In Situ Mounting Medium with DAPI (catalog #DUO82040), Duolink^®^ Blocking Solution (catalog #DUO82007), Duolink^®^ Antibody Diluent (DUO82008), Duolink^®^ In Situ Washing Buffer A (catalog #DUO82046), Duolink^®^ In Situ Washing Buffer B (catalog #DUO82048). Briefly, the samples were treated with 0.25% Triton X-100 (diluted in PBS) for 10 min at RT, and then blocked with Duolink^®^ blocking solution; samples were shaken at 37°C for 1 hr. Following incubation with designed primary antibodies (rabbit anti-vWF at 1:500, mouse anti-P-selectin at 1:500, and mouse anti-CD63 at 1:1000), the samples were incubated with the Duolink^®^ In Situ PLA^®^ anti-rabbit probe or anti-mouse probe, as appropriate. 1×Ligase (1 unit) added in 1×Ligation (39 units) was added to the sample prior to Duolink amplification. We used the spatial proximities of talin/α-catenin as a signal positive control and vWF-Rab5 as a negative control, respectively, in HUVECs for PLA (7). For a reagent negative control, normal mouse IgG and normal rabbit IgG were used as the primary antibodies. All samples were analyzed using an Olympus BX51 fluorescence microscope.

**Model for lipopolysaccharide (LPS)-induced endotoxemia in mice**

In this study, *EPAC1*-KO mice on a C57BL/6 background were derived as previously described (8). All mice were purpose-bred at UTMB and used between 8 and 12 weeks of age. *EPAC1*-KO mice were viable, fertile, and without overt abnormalities. We employed a mouse model of endotoxemia that consists of an intraperitoneal injection of a high dose of *E. coli* LPS (5 mg/kg) (*Escherichia coli* serotype O111:B4; Millipore Sigma) (9-11). LPS, which is derived from gram-negative bacteria, binds the LPS binding protein. In response to LPS, monocytes produce large quantities of proinflammatory cytokines, including TNFα (12). To measure vWF concentration in plasma, blood samples were collected 2 hrs after LPS treatment. After 24 hrs of LPS treatment, organs were dissected after euthanasia and perfusion via the right ventricle (13).

**Bleeding time, hemoglobin measurement, prothrombin time (PT), and activated partial thromboplastin time (aPTT) assays**

WT and *EPAC1*-KO mice were anesthetized with isoflurane. The distal 5 mm of the tail was clipped off with a scalpel. The bleeding tail was placed in 37°C PBS. The time until bleeding cessation was recorded as the bleeding time (14). Bleeding was also indirectly assessed by measuring the resultant hemoglobin content in the PBS solution. After centrifugation, blood cells were precipitated, and 1 mL RIPA buffer was added for cell lysis. Absorbance was read at 575 nm in a BioTek™ ELx808™ Absorbance Microplate Reader.

Plasma samples from citrated blood collected from WT and *EPAC1*-KO mice were used to determine PT and aPTT. Thromboplastin-D and aPTT-XL (ellagic acid activator) with CaCl_2_ were purchased from Thermo Fisher Scientific. The PT and aPTT were determined following the manufacturer’s instructions, and as described previously (5).

**Histopathology, immunofluorescence (IF) staining, and enzyme-linked immunosorbent assays (ELISA)**

Complete necropsies were performed on all experimentally-treated and control mice. Samples of brain, liver, and lung tissues were fixed in a 4% (vol/vol) neutral buffered solution of formaldehyde, embedded in paraffin, sectioned at 5-μm thickness, and processed by staining with hematoxylin and eosin for evaluation of histopathology. All IF reagents were obtained from Thermo Fisher Scientific. IF of vWF was performed in HUVECs. The sample was incubated with anti-vWF mouse monoclonal antibody (1:500). After 2 hrs, the samples were incubated with Alexa Fluor 488-conjugated goat anti-mouse IgG (1:2000) or Alexa Fluor 594-conjugated goat anti-rabbit IgG (1:1000) for 1 hr. A mouse monoclonal IgG served as a negative control. Nuclei were counterstained with 4′,6-diamidino-2-phenylindole (DAPI). Fluorescent images were taken and analyzed using an Olympus BX51-microscope, as described previously (7). IF of fibrin(ogen) was performed on mouse tissues as previously described (5).

Based on the IF images, quantitative analyses of Weibel-Palade bodies (WPBs) were performed using ImageJ software (National Institutes of Health, Bethesda, MD, USA) (15). Briefly, image thresholding was processed to create binary pictures revealing vWF positive puncta or cell nuclei, and then the size range of the puncta using the wand tool were determined. After the parameters were set, we used a particle analyzer to quantify the number of WPB puncta and cell nuclei. Normalization of the number of WPBs was done by dividing the number of WPBs by the number of cell nuclei in the same view.

Plasma samples from citrated blood collected from WT and *EPAC1*-KO mice were used for ELISAs to detect mouse vWF (ABclonal, Woburn, MA). The vWF concentrations in culture medium from HUVECs were detected using the human vWF ELISA Kit (Assaypro, MO). Standard curves were created by serial dilution of standard proteins provided in the kits. All ELISA plates were read at 450 nm, according to the manufacturer’s directions.

**Real-time quantitative polymerase chain reaction (qRT-PCR)**

Total RNA was prepared from tissue or cell samples using TRIzol™ (Thermo Fisher Scientific). Total RNA was quantitated using a NANODrop™ 2000 spectrophotometer. Complementary DNA was prepared using the iScript Reverse Transcription Supermix for qRT-PCR (Bio-Rad Laboratories, Inc., Hercules, CA), and qRT-PCR was carried out with primers for mouse vWF (forward: 5'-AAC AGA CGA TGG TGG ACT CAGC-3' and reverse: 5'-CGA TGG ACT CAC AGG AGC AAGT-3'), human eNOS (forward: 5'-GGC TCA GTT ACT GTC TAA GTG TTA GAA-3' and reverse: 5'-CCC TGG AGT CTT GTG TAG GAT AT-3'), human iNOS (forward: 5'-CCT GGC AGC CAT TTC AGA GGA G-3' and reverse: 5'-CCA GCC TCA AGT CTT ATT TCC TCA A-3'), and the iTaq Universal SYBR Green PCR Supermix (Bio-Rad Laboratories, Inc.). We normalized both mouse and human data by using two housekeeping genes, actin and glyceraldehyde-3-phosphate dehydrogenase (GAPDH), as references.

**Atomic force microscopy (AFM) to measure cell surface expression of target protein**

AFM is an advanced tool for studying biomechanical properties and has been used to determine the expression levels of cell surface proteins by measuring the binding affinity of specific protein–protein interactions, including antigen–antibody and receptor–ligand, with nano-force spectroscopy (16,17). In our study, anti-P-selectin or anti-CD63 antibodies were immobilized on polystyrene spheres attached to a colloidal cantilever. We measured the specific unbinding force during rupture of the interaction between the antigen (P-selectin or CD63) expressed in designated fields on the apical surface of living HUVECs and the antibody-coated AFM cantilever probe. Interactions between antibodies on the AFM cantilever and cell surface antigens cause large adhesion forces, which are quantified by the deflection signal during separation of the cantilever from the cell. By tracking the cantilever deflection and retraction cycle, the binding, stretching, and rupture of antibody–antigen complexes can be monitored in terms of the adhesive force changes on the cantilever over the distance traveled by the cantilever. We calculated the work required to break all interactive bonds between the cantilever and the EC, reflecting the quantity of antigen expression on the surface (16,17). As described previously (17), the biomechanical properties of P-selectin or CD63 at the cell surface were studied using an AFM system (Flex-AFM, Nanosurf AG, Liestal, Switzerland) that utilized relevant antibody-functionalized AFM probes. Colloidal cantilevers with a 5 μm polystyrene bead were used (SHOCON-G-PS, Applied NanoStructures, Mountain View, CA) to measure surface protein interaction forces (16,17). The cantilevers were functionalized by incubation with anti-P-selectin mAb (Thermo Fisher Scientific) or anti-CD63 mAb (Thermo Fisher Scientific) at 100 μg/ml in 0.1 M NaHCO_3_ buffer (pH 8.6) overnight at 4°C. Normal mouse IgG was used as negative control during calibration. Unbound proteins were rinsed off using PBS. The exposed surface of the bead was blocked by bovine serum albumin (Millipore Sigma) at 500 μg/ml in PBS. AFM imaging and measurements were generally taken within 1 hr after blocking. The spring constant of the cantilever was calibrated using the Sader method in air (18). The cantilever spring constant varied between 0.10-0.15 N/m. Force spectroscopy was done in static force mode operating on 25 μm^2^ areas on a living cell surface. The functionalized cantilever was manipulated into contact with the surface of a confluent monolayer of HUVECs. The maximum compression force was set to 150 pN. The contact time was kept constant at 500 msec before the cantilever was retracted at a constant pulling speed of 1 μm/s to measure the force-extension curve. Five cells per group were scanned, each with a different cantilever.

**Supplemental** **references**

1. Li, Y., Li, L., Dong, F., Guo, L., Hou, Y., Hu, H., Yan, S., Zhou, X., Liao, L., Allen, T. D., and Liu, J. U. (2015) Plasma von Willebrand factor level is transiently elevated in a rat model of acute myocardial infarction. *Exp Ther Med* **10**, 1743-1749

2. Gong, B., Shelite, T., Mei, F. C., Ha, T., Hu, Y., Xu, G., Chang, Q., Wakamiya, M., Ksiazek, T. G., Boor, P. J., Bouyer, D. H., Popov, V. L., Chen, J., Walker, D. H., and Cheng, X. (2013) Exchange protein directly activated by cAMP plays a critical role in bacterial invasion during fatal rickettsioses. *Proc Natl Acad Sci U S A* **110**, 19615-19620

3. Ye, N., Zhu, Y., Chen, H., Liu, Z., Mei, F. C., Wild, C., Cheng, X., and Zhou, J. (2015) Structure-Activity Relationship Studies of Substituted 2-(Isoxazol-3-yl)-2-oxo-N'-phenyl-acetohydrazonoyl Cyanide Analogues: Identification of Potent Exchange Proteins Directly Activated by cAMP (EPAC) Antagonists. *J Med Chem* **58**, 6033-6047

4. Wang, P., Luchowska-Stańska, U., van Basten, B., Chen, H., Liu, Z., Wiejak, J., Whelan, P., Morgan, D., Lochhead, E., Barker, G., Rehmann, H., Yarwood, S. J., and Zhou, J. (2020) Synthesis and Biochemical Evaluation of Noncyclic Nucleotide Exchange Proteins Directly Activated by cAMP 1 (EPAC1) Regulators. *J Med Chem* **63**, 5159-5184

5. He, X., Drelich, A., Yu, S., Chang, Q., Gong, D., Zhou, Y., Qu, Y., Yuan, Y., Su, Z., Qiu, Y., Tang, S. J., Gaitas, A., Ksiazek, T., Xu, Z., Zhou, J., Feng, Z., Wakamiya, M., Lu, F., and Gong, B. (2019) Exchange protein directly activated by cAMP plays a critical role in regulation of vascular fibrinolysis. *Life Sci* **221**, 1-12

6. Drelich, A., Judy, B., He, X., Chang, Q., Yu, S., Li, X., Lu, F., Wakamiya, M., Popov, V., Zhou, J., Ksiazek, T., and Gong, B. (2018) Exchange Protein Directly Activated by cAMP Modulates Ebola Virus Uptake into Vascular Endothelial Cells. *Viruses* **10**, 563

7. Liu, Y., Xiao, J., Zhang, B., Shelite, T. R., Su, Z., Chang, Q., Judy, B., Li, X., Drelich, A., Bei, J., Zhou, Y., Zheng, J., Jin, Y., Rossi, S. L., Tang, S. J., Wakamiya, M., Saito, T., Ksiazek, T., Kaphalia, B., and Gong, B. (2020) Increased talin-vinculin spatial proximities in livers in response to spotted fever group rickettsial and Ebola virus infections. *Lab Invest*, 1-12

8. Yan, J., Mei, F. C., Cheng, H., Lao, D. H., Hu, Y., Wei, J., Patrikeev, I., Hao, D., Stutz, S. J., Dineley, K. T., Motamedi, M., Hommel, J. D., Cunningham, K. A., Chen, J., and Cheng, X. (2013) Enhanced leptin sensitivity, reduced adiposity, and improved glucose homeostasis in mice lacking exchange protein directly activated by cyclic AMP isoform 1. *Mol Cell Biol* **33**, 918-926

9. Fink, M. P. (2014) Animal models of sepsis. *Virulence* **5**, 143-153

10. Catorce, M. N., and Gevorkian, G. (2016) LPS-induced Murine Neuroinflammation Model: Main Features and Suitability for Pre-clinical Assessment of Nutraceuticals. *Curr Neuropharmacol* **14**, 155-164

11. Schabbauer, G., Tencati, M., Pedersen, B., Pawlinski, R., and Mackman, N. (2004) PI3K-Akt pathway suppresses coagulation and inflammation in endotoxemic mice. *Arterioscler Thromb Vasc Biol* **24**, 1963-1969

12. van der Bruggen, T., Nijenhuis, S., van Raaij, E., Verhoef, J., and van Asbeck, B. S. (1999) Lipopolysaccharide-induced tumor necrosis factor alpha production by human monocytes involves the raf-1/MEK1-MEK2/ERK1-ERK2 pathway. *Infect Immun* **67**, 3824-3829

13. Gage, G. J., Kipke, D. R., and Shain, W. (2012) Whole animal perfusion fixation for rodents. *J Vis Exp* **30**, 3564

14. Jirouskova, M., Shet, A. S., and Johnson, G. J. (2007) A guide to murine platelet structure, function, assays, and genetic alterations. *J Thromb Haemost* **5**, 661-669

15. Schindelin, J., Rueden, C. T., Hiner, M. C., and Eliceiri, K. W. (2015) The ImageJ ecosystem: An open platform for biomedical image analysis. *Mol Reprod Dev* **82**, 518-529

16. Mostowy, S., Janel, S., Forestier, C., Roduit, C., Kasas, S., Pizarro-Cerda, J., Cossart, P., and Lafont, F. (2011) A role for septins in the interaction between the Listeria monocytogenes INVASION PROTEIN InlB and the Met receptor. *Biophys J* **100**, 1949-1959

17. Gong, B., Ma, L., Liu, Y., Gong, Q., Shelite, T., Bouyer, D., Boor, P. J., Lee, Y. S., and Oberhauser, A. (2012) Rickettsiae induce microvascular hyperpermeability via phosphorylation of VE-cadherins: evidence from atomic force microscopy and biochemical studies. *PLoS Negl Trop Dis* **6**, e1699

18. Xie, H., Yin, M., Rong, W., and Sun, L. (2014) In situ quantification of living cell adhesion forces: single cell force spectroscopy with a nanotweezer. *Langmuir* **30**, 2952-2959

**Supplemental Figures:**

**Figure S1:** (A) Immunofluorescence staining using rabbit anti-CD31 (red) on adjacent-level sections from the mouse lungs as in Figure 1D. Nuclei are counterstained blue with DAPI. (B) Immunofluorescence staining of vWF (green), P-selectin (red), and CD63 (red), respectively, in human umbilical vein endothelial cell (HUVEC) cultures. Nuclei are counterstained blue with DAPI. Scalebars, 20 µm.

**Figure S2**: **Changes in the coagulative state of LPS-treated *EPAC1*-KO mice.** (A) Prothrombin time (PT) and (B) activated partial thromboplastin time (aPTT) of LPS-treated WT and *EPAC1*-KO mice. The differences in PT and aPTT between these two groups were not statistically significant (n=5).

**Figure S3**: **The effect of NY173 and I942 on HUVEC viability and vWF secretion.** (A) The viability of HUVECs was detected using the MTT Cell Proliferation Assay kit from Cayman Chemical. HUVECs were incubated with different concentrations of NY173 (1, 2, 3, or 5 μM) or I942 (5, 10, 30, or 50 μM) for 24 h. 0.1% DMSO-treated HUVECs served as controls. 200 μM H_2_O_2_-treated HUVECs served as positive control. n=3 for each group. (B) The viability of brain microvascular ECs (BMECs) was also detected using the MTT assay kit.

**Figure S4**: **Reverse transcription-quantitative polymerase chain reaction (RT-qPCR) analysis of iNOS mRNA expression in HUVECs.** The difference in iNOS mRNA expression among the rTNFα-only group, NY173 + rTNFα treated-group, and the NY173 + rTNFα + IRS treated group was not statistically significant. n = 4 for each group**.**

**Figure S1**


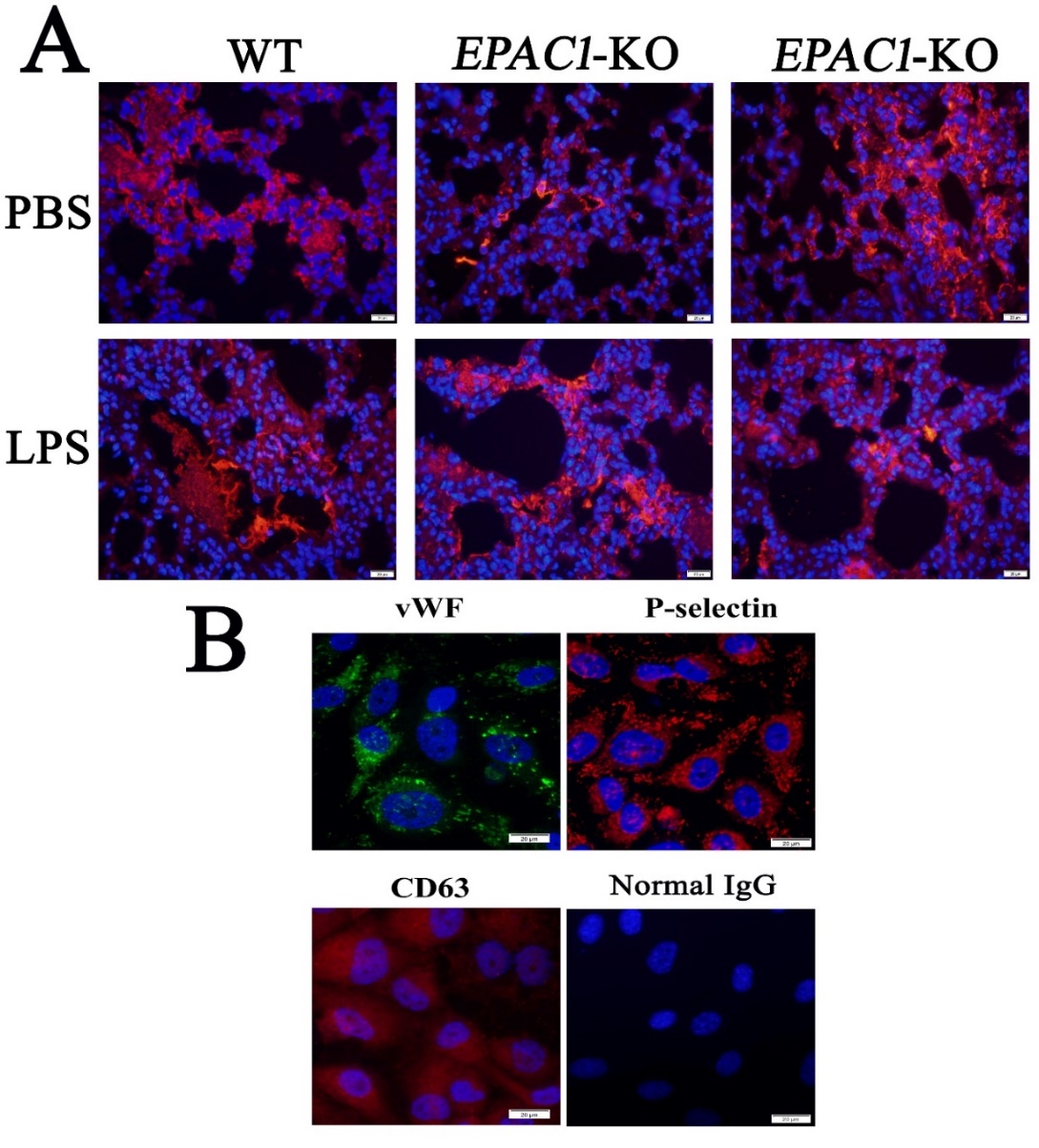


**Figure S2**


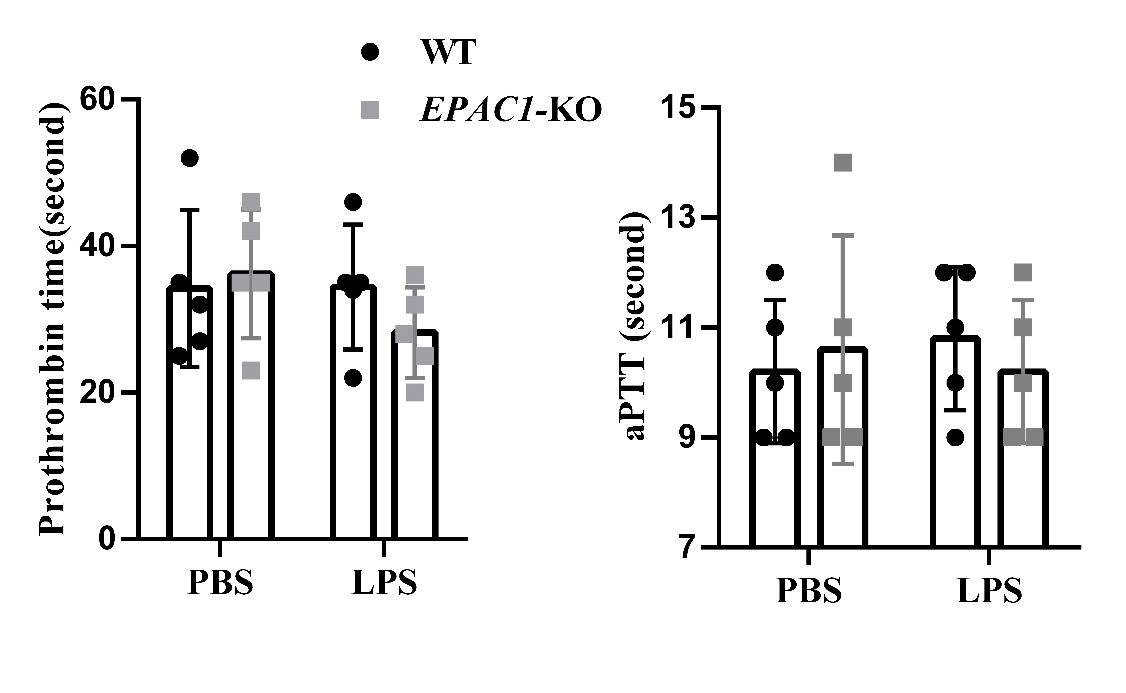


**Figure S3**


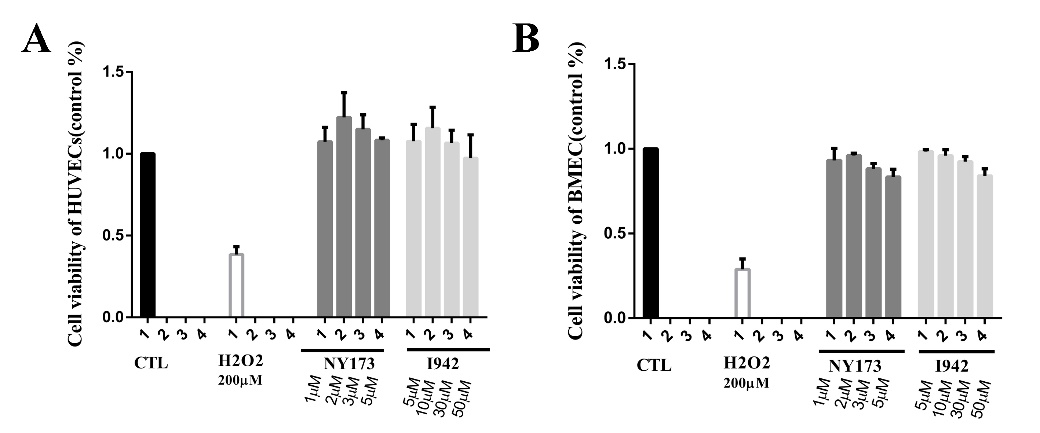


**Figure S4**


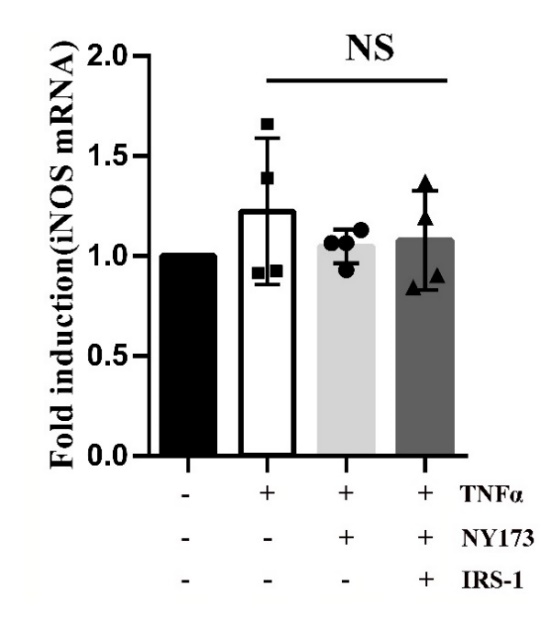

Supplement: Supporting information [file mmc1.docx]
